# AICAR Inhibits Insulin-Stimulated Glucose Uptake in 3T3-L1 Adipocytes via an AMPK-Independent, ZMP-Dependent Mechanism

**DOI:** 10.3390/cells14221811

**Published:** 2025-11-18

**Authors:** Yazeed Alshuweishi, Fatmah Binzomah Alghamdi, Kieran Patrick, Ian P. Salt

**Affiliations:** 1 School of Molecular Biosciences, College of Veterinary, Medical and Life Sciences, University of Glasgow, Glasgow G12 8QQ, UK; yalshuweishi@ksu.edu.sa (Y.A.); fsaalghamdi7@kau.edu.sa (F.B.A.);; 2 Department of Clinical Laboratory Sciences, King Saud University, Riyadh 11433, Saudi Arabia; 3 Department of Clinical Pharmacology, Faculty of Medicine, King Abdulaziz University, Jeddah 21589, Saudi Arabia

**Keywords:** AMPK, adipocyte, glucose uptake, GLUT4

## Abstract

**Highlights:**

**What are the main findings?**

- Sustained incubation with AICAR inhibits insulin-stimulated glucose uptake in adipocytes in an AMPK-independent but ZMP-dependent manner
- Sustained incubation with AICAR inhibits insulin-stimulated ERK1/2 phosphorylation in adipocytes in a ZMP-dependent manner

**What are the implications of the main findings?**

- Unlike muscle, sustained AMPK activation in adipocytes does not alter levels of glucose transporters.
- Adenine nucleotides regulate both insulin-stimulated glucose uptake and ERK1/2 phosphorylation in adipocytes.

**Abstract:**

AMP-activated protein kinase (AMPK) is activated by reduced cellular energy charge and mimics the action of insulin in muscle by stimulating increased trafficking of GLUT4 to the plasma membrane. In contrast, we have previously reported that short-term activation of AMPK in adipocytes has no effect on glucose uptake. Whether prolonged AMPK activation influences adipocyte glucose uptake remains poorly characterised. To investigate the effect of sustained AMPK activation on glucose uptake in adipocytes, glucose uptake and insulin signalling were assessed in 3T3-L1 adipocytes stimulated with AICAR and 991, which activate AMPK by different mechanisms, for 24 h. Furthermore, glucose uptake and GLUT4 levels were assessed in adipocytes or adipose tissue from mice lacking AMPKα1 as a model of prolonged AMPK downregulation. AICAR, but not 991, markedly inhibited insulin-stimulated glucose uptake in 3T3-L1 adipocytes. This effect of AICAR was associated with impaired trafficking of GLUT4 to the plasma membrane but did not alter cellular GLUT4 levels or insulin signalling via AKT. The effect of AICAR did, however, require phosphorylation to the nucleotide ZMP and was associated with altered insulin-stimulated MEK1/2-ERK1/2 phosphorylation. Sustained AMPK downregulation had no effect on adipocyte glucose uptake or GLUT4 levels. Taken together, these data demonstrate that sustained changes in AMPK activity do not alter adipocyte glucose uptake. Furthermore, AICAR reduces insulin-stimulated GLUT4 translocation and glucose uptake in adipocytes by a mechanism that is independent of AMPK but requires phosphorylation of AICAR to ZMP.

## 1. Introduction

One key action of insulin is to stimulate glucose uptake into striated muscle and adipocytes via the glucose transporter GLUT4, thereby permitting increased synthesis of glycogen and triglycerides, respectively [1,2]. Insulin stimulates activation of the serine/threonine protein kinase AKT (protein kinase B), which subsequently phosphorylates and inhibits the Rab GTPase activating proteins TBC1D1 and AS160 (TBC1D4) [1,2]. This increases GTP-bound Rab levels, which facilitate translocation of GLUT4 from an intracellular compartment to the plasma membrane, thereby increasing glucose uptake [1,2].

AMP-activated protein kinase (AMPK) is a heterotrimeric Ser/Thr protein kinase activated when cellular energy charge is depleted due to insufficient ATP production or excessive ATP consumption [2,3,4]. AMPK subsequently phosphorylates substrate proteins that act to normalise cellular ATP levels, either by increasing ATP production or suppressing ATP consumption [2,3,4]. AMPK can therefore be activated by any stimulus that impairs mitochondrial ATP production, including metformin, resveratrol, dinitrophenol and canagliflozin [5]. Other activators have been developed that can stimulate AMPK without altering adenine nucleotide ratios. The first of these was the ribonucleoside AICAR (5-aminoimidazole-4-carboxamide ribonucleoside), which is taken up by cells and phosphorylated by adenosine kinase to the nucleotide ZMP, which can mimic AMP and thereby activate AMPK [6,7]. ZMP, however, will influence other AMP-sensitive enzymes, including fructose 1,6-bisphosphatase (FBP1) and glycogen phosphorylase (GP) [8,9]. More specific AMPK activators have since been developed that allosterically activate AMPK by binding a site at the interface of the α catalytic and β regulatory subunits, including A769662 and 991, although both of these have also been demonstrated to have AMPK-independent actions [10,11,12].

One key metabolic action of AMPK in muscle is to mimic insulin and stimulate glucose uptake via increased trafficking of GLUT4 [2,13]. Originally, AMPK was proposed to underlie the GLUT4-mediated increase in glucose uptake during muscle contraction [14,15], as activated AMPK mimics insulin-stimulated AKT by directly phosphorylating AS160 and TBC1D1 [16]. It is currently thought, however, that AMPK does not underlie contraction-mediated glucose uptake in muscle, yet does increase insulin sensitivity and glycogen synthesis in recovery after exercise [13,17]. In adipocytes, however, the role of AMPK in the regulation of glucose uptake is far less well characterised. Early studies reported that AICAR rapidly reduced insulin-stimulated glucose uptake in both 3T3-L1 adipocytes and isolated rat adipocytes [18,19]. This AICAR-mediated inhibition of insulin-stimulated glucose uptake was reported to be AMPK-dependent and associated with increased AS160 T642 phosphorylation in rat adipocytes [20], yet a recent study showed no difference in AS160 T642 phosphorylation in response to AICAR [11]. Furthermore, in that study, the effect of AICAR and direct AMPK activators on glucose uptake was compared, in which AICAR and A769662 inhibited insulin-stimulated glucose uptake in several different adipocyte models, yet the more potent and selective AMPK activator 991 had no effect on glucose uptake, despite robustly activating AMPK [11]. The mechanism by which AICAR reduces insulin-stimulated glucose uptake, therefore, remains unclear.

The studies indicated above have used relatively short-term stimulation with AICAR or other AMPK activators of up to 2 h. In muscle, sustained stimulation with AICAR for 18 h increased expression of GLUT4 in mice [21], an effect reported to be due to AMPK-mediated phosphorylation of GLUT4 enhancer factor (GEF) [22] and/or histone deacetylase-5 (HDAC5) [23], yet the effect of prolonged stimulation of adipocytes with AMPK activators has not been assessed. Furthermore, adipocyte glucose transport has not been directly assessed in isolated adipocytes from AMPK-deficient mice, although unaltered 2-deoxyglucose uptake in vivo in gonadal WAT as well as isolated fat pads has been reported in mice lacking AMPK subunits [24,25].

Therefore, to investigate the effect of sustained AMPK activation on glucose uptake in adipocytes, glucose uptake and insulin signalling were assessed in 3T3-L1 adipocytes stimulated with AICAR and 991, which activate AMPK by different mechanisms, for 24 h. Furthermore, to investigate the effect of sustained reduced AMPK activity, glucose uptake and GLUT4 levels were assessed in adipocytes or adipose tissue from mice lacking AMPKα1 [26,27], the catalytic isoform responsible for the majority of total cellular AMPK activity in adipocytes [18,28,29].

## 2. Materials and Methods

### 2.1. Materials

The 3T3-L1 preadipocytes were obtained from ATCC. The 3T3-L1 preadipocytes stably expressing HA-GLUT4-GFP (3T3-HA-GLUT4-GFP) were generously provided by Prof. Gwyn Gould (University of Strathclyde) and were generated by lentiviral infection using plasmids originally provided by Prof. Cynthia Mastick (University of Nevada) and have been described previously [30]. The 2-deoxyglucose, cytochalasin B, IBMX (3-Isobutyl-1-methylxanthine), silicone oil, dexamethasone and porcine insulin were purchased from Sigma Aldrich, Gillingham, UK. Troglitazone, 5-iodotubercidin, PD184352 and ABT-702 were obtained from Tocris, Bristol, UK. AICAR was purchased from Toronto Research Chemicals, Toronto, ON, Canada. 991 was synthesised by MRC Technology, Stevenage, UK. [^3^H]2-deoxyglucose (NET328) was from Revvity, Oxford, UK. Mouse anti-HA antibodies (#MMS-101P) were from Biolegend, San Diego, CA, USA. Rabbit anti-ACC (acetyl-CoA carboxylase, #3662), anti-phospho-ACC S79 (#3661), anti-phospho-AKT S473 (#4058), anti-phospho-AKT T308 (#13038), anti-phospho-AS160 T642 (#8881), anti-ERK1/2 (extracellular signal-regulated kinase, #9102), anti-HK2 (hexokinase-2, #2867) and anti-phospho-MEK1/2 S217/220 (mitogen-activated protein kinase kinase, #9154) antibodies and mouse anti-AKT (#2920), anti-phospho-ERK1/2 T202/Y204 (#9106) and anti-MEK1/2 (#4694) antibodies were from Cell Signaling Technology, Leiden, The Netherlands. Rabbit anti-GLUT4 (ab654) antibodies were from Abcam, Cambridge, UK. Rabbit anti-AS160 antibodies (#07-741) were obtained from Merck, Darmstadt, Germany. REVERT total protein stain, IRdye680 or 800-labelled donkey anti-mouse IgG (#926-32212) and anti-rabbit IgG (#926-68023 and #926-32213) antibodies were from LI-COR Biosciences, Cambridge, UK. AlexaFluor 568-linked goat anti-mouse IgG antibodies (#A-11004) and DMEM (#41965039) were from Thermo Fisher Scientific, Paisley, UK.

### 2.2. Mouse Epididymal Adipose Tissue and Isolation of Adipocytes

Wild-type Sv129 (WT) or mice lacking AMPKα1 (AMPKα1 KO) were kindly supplied by Prof. Erik Richter (University of Copenhagen) and Prof. Benoit Viollet (Institut Cochin, Paris, France). The generation of these animals has been described before [26]. Mice were housed at the Central Research Facility at the University of Glasgow and kept on 12 h cycles of light and dark and at ambient temperature. All animal experiments were performed in accordance with the United Kingdom Home Office Legislation under the Animals (Scientific Procedure) Act 1986 (project licences 60/4114, 70/8572 and PP1756142 which were approved by the Glasgow University Animal Welfare and Ethical Review Board in 2010, 2015 and 2020, respectively) and guidelines from Directive 2010/63/EU of the European Parliament on the protection of animals used for scientific purposes. Mice (20-week-old) were euthanised (Schedule 1 procedure) and the gonadal adipose tissues were rapidly excised. For analysis of GLUT4 mRNA and protein levels, epididymal adipose tissue was immediately snap-frozen in liquid nitrogen. For [^3^H]2-deoxyglucose uptake assays, female gonadal adipose tissue was collected in KRH buffer (128 mM NaCl, 4.7 mM KCl, 5 mM NaH_2_PO_4_, 1.2 mM MgSO_4_, 20 mM HEPES-NaOH, 1.5 mM CaCl_2_, 1% (*w*/*v*) BSA, 10 µM adenosine, 3 mM glucose) at 37 °C. Adipose tissue was minced and digested in 4 vol KRH buffer containing type I collagenase (1 mg/mL, Worthington, Reading, UK) with shaking at 37 °C for 30–40 min. Adipocytes were washed by flotation in KRH (four times). Primary mouse adipocytes were used directly after isolation.

### 2.3. Culture and Differentiation of 3T3-L1 Adipocytes

The 3T3-L1 and 3T3-HA-GLUT4-GFP preadipocytes were maintained in DMEM supplemented with 10% (*v*/*v*) newborn calf serum at 37 °C in a 10% CO_2_ atmosphere. Two days after confluence, differentiation of cells was started by culture in DMEM supplemented with 10% (*v*/*v*) foetal calf serum (FCS), 0.5 mM IBMX, 0.25 µM dexamethasone, 1 µg/mL insulin and 5 µM troglitazone for 3 days. Medium was replaced with DMEM supplemented with FCS, troglitazone and insulin for a further 3 days, then DMEM supplemented with FCS alone for 2–6 days, such that adipocytes were used for experimentation 8–12 days post-differentiation.

### 2.4. Preparation of 3T3-L1 Adipocyte or Adipose Tissue Lysates/Homogenates

3T3-L1 adipocytes were incubated in DMEM supplemented with 2% (*v*/*v*) FCS in the presence or absence of compounds for 24 h prior to stimulation in the presence or absence of insulin (100 nM) for 15 min. Medium was removed and cells lysed in RIPA buffer (50 mM Tris-HCl, pH 7.4 at 4 °C, 50 mM NaF, 1 mM Na4P_2_O_7_, 1 mM EDTA, 1 mM EGTA, 1% (*v*/*v*) NP-40, 250 mM mannitol, 1 mM dithiothreitol, 1 mM Na_3_VO_4_, 0.1 mM benzamidine, 0.1 mM phenylmethylsulphonyl fluoride, 5 µg/mL soybean trypsin inhibitor, 0.1% (*w*/*v*) SDS, 0.1% (*w*/*v*) sodium deoxycholate). Mouse adipose tissues were homogenised in 4 vol RIPA buffer at 4 °C for 3 min in a TissueLyser using metallic beads. Homogenates were transferred to a microcentrifuge tube and placed on ice for 20 min. Lysates/homogenates were centrifuged (21,910× *g*, 10 min, 4 °C) and supernatants stored at -20 °C prior to use.

### 2.5. SDS-PAGE and Immunoblotting

Equal amounts of cell lysate/tissue homogenate protein, as determined by BCA (bicinchoninic acid) assay, were resolved on polyacrylamide gels and transferred onto nitrocellulose using a Mini Protein trans blot apparatus (Bio-Rad Laboratories Ltd, Watford, UK). Membranes were stained with REVERT total protein stain according to the manufacturer’s instructions, destained, washed in Tris-buffered saline (TBS, 20 mM Tris-HCl, pH 7.5, 137 mM NaCl) and blocked in TBS supplemented with 5% (*w*/*v*) milk powder for 30 min. Membranes were washed in TBST (TBS supplemented with 0.1% (*v*/*v*) Tween-20) and incubated overnight with primary antibodies in TBST supplemented with 5% (*w*/*v*) BSA at 4 °C. Membranes were washed with TBST, then incubated in appropriate IRdye-conjugated secondary donkey antibodies in the dark for 1 h at room temperature. Membranes were washed in TBST and immunoreactive bands visualised using a LI-COR Odyssey infrared imaging system. Band densities and total protein staining were quantified using LI-COR Empiria Studio 3.2 software.

### 2.6. Uptake of [^3^H]2-Deoxyglucose in 3T3-L1 Adipocytes and Mouse Adipocytes

Glucose uptake was measured by the uptake of [^3^H]2-deoxyglucose as described previously [11]. Briefly, cells were stimulated as described in Krebs-Ringer-phosphate (KRP (128 mM NaCl, 4.7 mM KCl, 5 mM NaH_2_PO_4_ (pH 7.4), 1.2 mM MgSO_4_, 2.5 mM CaCl_2_, 1% (*w*/*v*) BSA) buffer. Transport was initiated by adding [^3^H]-2deoxyglucose (50 μM, 1 μCi/mL). After 3 min, cells were rapidly washed in ice-cold PBS, air-dried, and solubilised in 1% (*v*/*v*) Triton X-100. For [^3^H]2-deoxyglucose uptake in female gonadal adipocytes (10% cytocrit in KRH buffer), cells were stimulated in the presence or absence of 10 nM insulin for 30 min prior to the addition of [^3^H]-2deoxyglucose (10 µM, 0.2 µCi/mL). After 10 min, adipocytes were separated by centrifugation through silicone oil. Adipocytes were collected and solubilised in 1% (*v*/*v*) Triton X-100. Adipocyte-associated ^3^H was determined by liquid scintillation spectrophotometry. Non-specific cell-associated radioactivity was determined in parallel incubations performed in the presence of 10 μM cytochalasin B.

### 2.7. RNA Extraction and Gene Expression Analysis in 3T3-L1 Adipocytes and Mouse Epididymal Adipose Tissue

RNA was extracted from 3T3-L1 adipocytes and mouse epididymal adipose tissue using a RNeasy kit (Qiagen, Manchester, UK). In the case of mouse epididymal adipose tissue, this included initial homogenisation of 50 mg adipose tissue with 1 mL Qiazol and two 5 mm stainless steel balls in a TissueLyser LT (Qiagen) for 10 min. Adipose tissue homogenates were then incubated at room temperature for 5 min prior to adding chloroform, shaken vigorously for 15 sec and incubated again at room temperature for 3 min. Beads were then removed and samples centrifuged at 12,000× *g* for 15 min at 4 °C. Ethanol (1 vol, 70% (*v*/*v*)) was added to the upper aqueous phase and RNA subsequently extracted with a RNeasy kit. Between 400–1000 ng RNA was reverse-transcribed using a High-Capacity cDNA Reverse Transcription kit (Thermo Fisher) and qPCR using Taqman Assays on Demand and master mix (Thermo Fisher) performed with an Applied Biosystems ABI-PRISM 7900HT Sequence Detection System (Thermo Fisher). The following TaqMan gene expression assays were used: *Tbp* (Mm01277042, encoding TATA-binding protein), *Slc2a1* (Mm00441480, encoding GLUT1) and *Slc2a4* (Mm00436615, encoding GLUT4). All data were normalised to levels of *Tbp*, and relative quantification was calculated as 2^−ΔCt^.

### 2.8. Fluorescence Confocal Microscopy of 3T3-HA-GLUT4-GFP Cells

Cells were differentiated on glass cover slips and stimulated as indicated. Cells were fixed in 4% (*w*/*v*) paraformaldehyde for 30 min and washed with PBS and incubated in PBS supplemented with 2% (*w*/*v*) BSA and 20 mM glycine (BSA/GLY) for 20 min. After this, cells were incubated with mouse anti-HA antibodies (1:500 in BSA/GLY) for 45 min, washed and incubated with AlexaFluor 568-conjugated goat anti-mouse IgG antibodies (1:200 in BSA/GLY) for 30 min. Cells were subsequently washed and embedded in Immumount on slides. Confocal microscopy of 3T3-HA-GLUT4-GFP cells was performed on a Zeiss LSM5 Exciter equipped with a 63X objective. Images were captured and processed using Zeiss LSM Image Browser 4.2.0.121 software (Carl Zeiss microscopy Ltd, Cambridge, UK). For each experiment, 10–20 random fields were captured. Quantification was performed using ImageJ 1.54, where regions of interest were defined from the GFP channel to delineate individual cell boundaries. Background-subtracted integrated fluorescence intensities were obtained for GFP (representing total cellular GLUT4) and HA (representing plasma membrane-localised GLUT4). A surface-to-total ratio (HA/GLUT4) was calculated for each cell.

### 2.9. Statistical Analysis

Data are presented as mean ± SEM. Unless otherwise described, data with one variable were analysed using Student’s *t*-tests and data with two variables by 2-way ANOVA with Šídák’s multiple comparisons tests. *p* < 0.05 was considered statistically significant. All statistical analyses were performed using Graphpad Prism 10 software.

## 3. Results

### 3.1. AICAR but Not 991 Inhibits Insulin-Stimulated Glucose Uptake Without Influencing GLUT4 or HK2 Levels

To examine the effects of prolonged incubation with AICAR or C991 on AMPK activity in 3T3-L1 adipocytes, cells were incubated in the presence or absence of AICAR (1 mM) or C991 (5 µM) for 24 hr, and the phosphorylation of the AMPK substrate ACC S79 was determined by immunoblotting.

**Figure 1:**
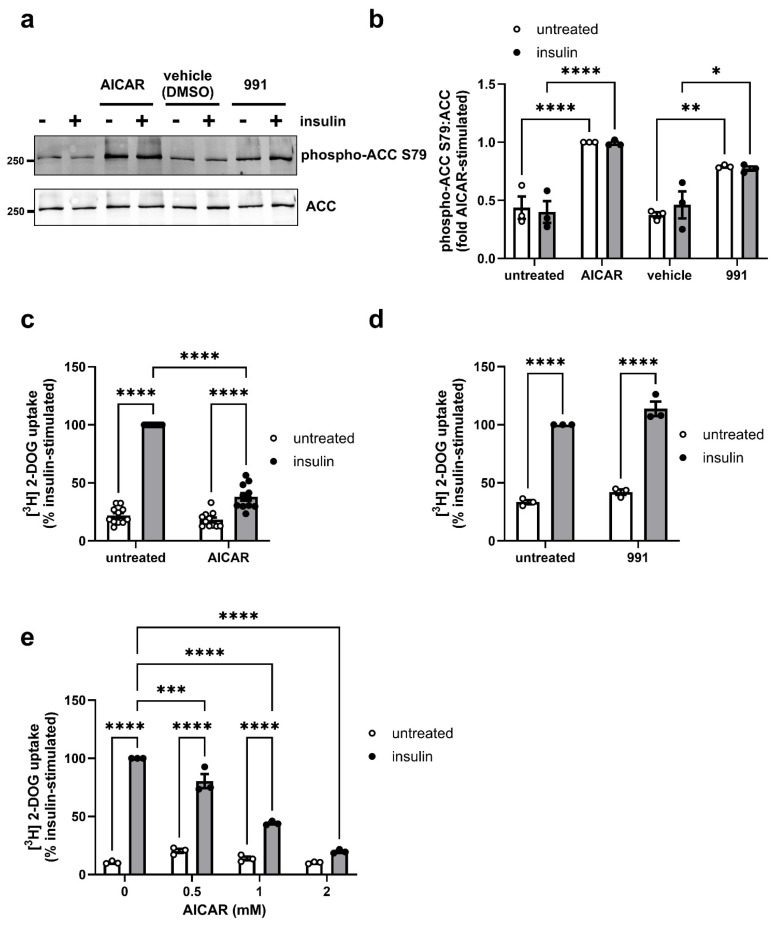
Effect of AICAR and 991 on ACC phosphorylation and [^3^H]2-deoxyglucose uptake in 3T3-L1 adipocytes. 3T3-L1 adipocytes were stimulated in the presence or absence of (**a**–**c**) 1 mM AICAR or (**a**,**b**,**d**) 5 µM 991 or (**e**) 0–2 mM AICAR for 24 h prior to the presence or absence of insulin (100 nM, 15 min). DMSO was used as a vehicle control for 991. (**a**,**b**) Lysates were prepared and immunoblotted with anti-ACC or anti-phospho-ACC S79 antibodies. (**a**) Representative immunoblots are shown with indicated molecular masses in kDa. (**b**) Quantification of ACC S79 phosphorylation normalised to ACC from three independent experiments. (**c**–**e**) % insulin-stimulated [^3^H]-2-deoxyglucose ([^3^H]2-DOG) uptake from (**c**) 11 or (**d**,**e**) 3 independent experiments (* *p* < 0.05, ** *p* < 0.01, *** *p* < 0.001, **** *p* < 0.0001).

ACC phosphorylation was significantly increased to a similar extent after incubation with either activator (Figure 1a,b). Insulin had no effect on ACC phosphorylation. To determine whether long-term AMPK activation influenced glucose uptake in 3T3-L1 adipocytes, cells were incubated in the presence and absence of a saturating concentration of insulin (100 nM, 15 min, Supplemental Figure S1a) after preincubation with AICAR (1 mM) or 991 (5 µM) for 24 h, and [^3^H]2-deoxyglucose uptake was assessed. AICAR significantly inhibited insulin-stimulated glucose uptake by 62 ± 3% without altering basal uptake (Figure 1c). In contrast, 991 had no effect on basal or insulin-stimulated 2-deoxyglucose uptake (Figure 1d). The inhibitory effect of AICAR on insulin-stimulated glucose uptake was concentration-dependent, with complete inhibition by 2 mM AICAR (Figure 1e and Supplemental Figure S1b).

**Figure 2:**
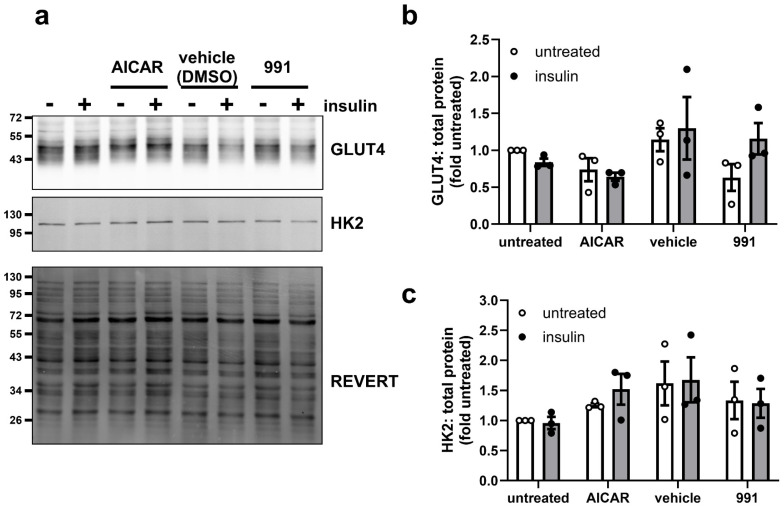
Effect of AICAR and 991 on GLUT4 and HK2 levels in 3T3-L1 adipocytes. 3T3-L1 adipocytes were stimulated in the presence or absence of 1 mM AICAR or 5 µM 991 for 24 h prior to incubation in the presence or absence of insulin (100 nM, 15 min). DMSO was used as a vehicle control for 991. Lysates were prepared and immunoblotted with anti-GLUT4 or anti-HK2 antibodies. (**a**) Representative immunoblots are shown with the indicated molecular masses in kDa. Quantification of (**b**) GLUT4 or (**c**) HK2 levels normalised to REVERT total protein stain from three independent experiments.

Given the marked inhibition of insulin-stimulated 2-deoxyglucose uptake by AICAR, the abundance of GLUT4 was examined. There were no observed changes in the levels of GLUT4 in response to incubation with AICAR or 991 for 24 h (Figure 2a,b). As 2-deoxyglucose uptake assays reflect glucose transport and hexokinase (HK)-mediated phosphorylation to 2-deoxyglucose-6-phosphate, levels of the principal adipose tissue HK isoform HK2 [31] were also assessed. Neither AICAR nor 991 had any effect on levels of HK2 (Figure 2a,c). Furthermore, incubation with either AICAR or 991 for 48 h had no effect on expression of *Slc2a4* mRNA (encoding GLUT4) in 3T3-L1 adipocytes (Supplemental Figure S1b). As GLUT1, encoded by *Slc2a1*, has been reported to contribute to insulin-stimulated glucose uptake in 3T3-L1 adipocytes [32], expression of *Slc2a1* was also examined. AICAR increased *Slc2a1* levels in 3T3-L1 adipocytes, whereas incubation with 991 for 48 h had no effect (Supplemental Figure S1c).

### 3.2. AICAR but Not 991 Reduces Insulin-Stimulated GLUT4 Translocation in 3T3-L1 Adipocytes

**Figure 3:**
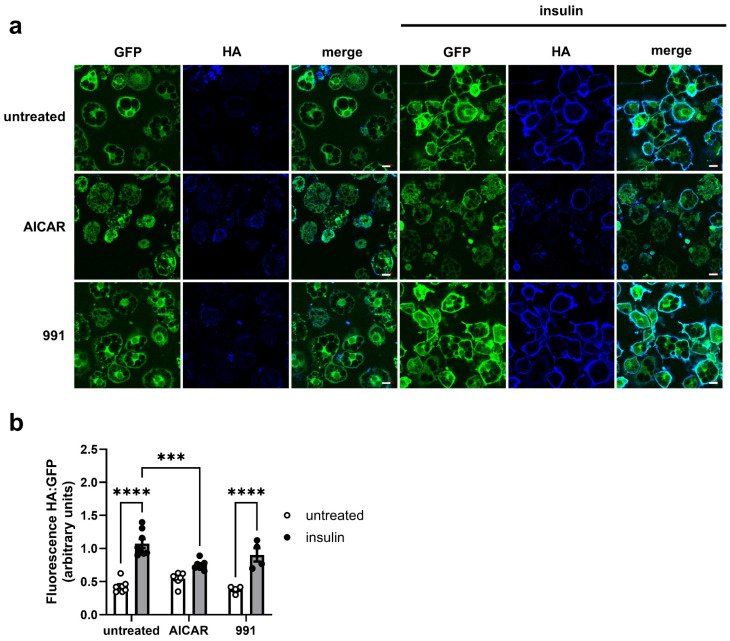
Effect of AICAR and 991 on insulin-stimulated GLUT4 translocation in 3T3-L1 adipocytes. 3T3-HA-GLUT4-GFP adipocytes were stimulated in the presence or absence of 1 mM AICAR or 5 µM 991 for 24 h prior to the presence or absence of insulin (100 nM, 30 min) in serum-free media. Cells were then washed, fixed and stained for cell surface GLUT4 with anti-HA antibodies. (**a**) Representative fields of cells showing GFP in green and HA staining in blue (white scale bar represents 10 µm). (**b**) Quantification of anti-HA staining relative to GFP from 4–7 independent experiments (*** *p* < 0.001, **** *p* < 0.0001).

To examine whether the inhibitory effects of prolonged AICAR treatment on insulin-stimulated glucose uptake were associated with impaired insulin-stimulated GLUT4 translocation, translocation of GLUT4 was assessed in 3T3-L1 adipocytes stably expressing HA-GLUT4-GFP, which contains an exofacial HA epitope, allowing assessment of plasma membrane GLUT4 in non-permeabilised cells [30,33]. Insulin (100 nM, 30 min) led to a ~2.5-fold increase in GLUT4 at the cell surface (Figure 3). Preincubation for 24 h with 1 mM AICAR significantly attenuated insulin-stimulated GLUT4 translocation, whereas preincubation with 991 had no effect (Figure 3).

### 3.3. AICAR and 991 Have No Effect on Insulin-Stimulated AKT Signalling

**Figure 4:**
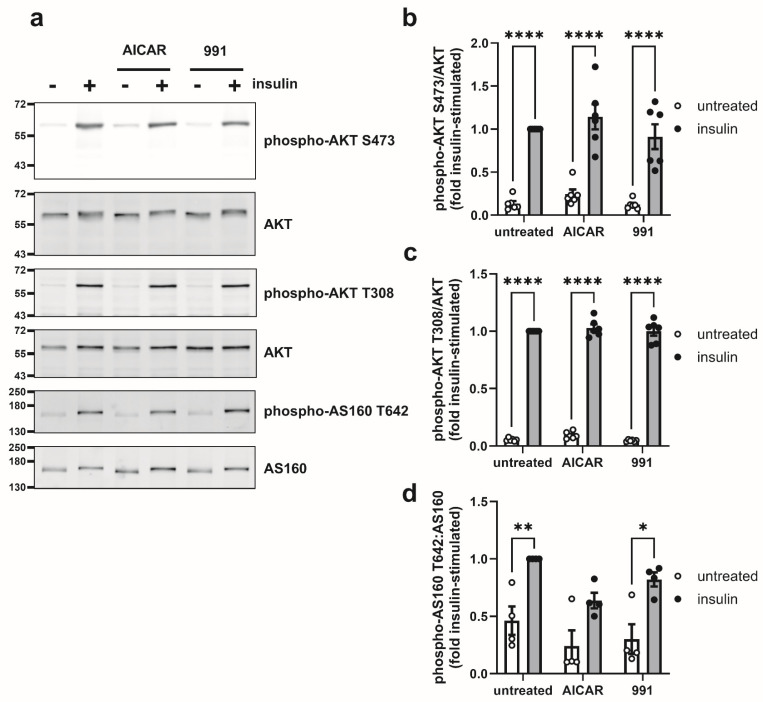
Effect of AICAR and 991 on AKT and AS160 phosphorylation in 3T3-L1 adipocytes. 3T3-L1 adipocytes were stimulated in the presence or absence of 1 mM AICAR or 5 µM 991 for 24 h prior to the presence or absence of insulin (100 nM, 15 min). Lysates were prepared and immunoblotted with the antibodies indicated. (**a**) Representative immunoblots are shown with the indicated molecular masses in kDa. (**b**–**d**) Quantification of (**b**) AKT S473 phosphorylation normalised to AKT, (**c**) AKT T308 phosphorylation normalised to AKT or (**d**) AS160 T642 phosphorylation relative to AS160 from (**b**,**c**) six or (**d**) four independent experiments (* *p* < 0.05, ** *p* < 0.01, **** *p* < 0.0001).

To determine whether AICAR suppressed insulin-stimulated glucose uptake by inhibiting the AKT-AS160 signalling pathway necessary for GLUT4 trafficking, the effect of AICAR and 991 was assessed on AKT phosphorylation at S473 and T308, as well as phosphorylation of the AKT substrate AS160 at T642 by immunoblotting. Insulin significantly stimulated phosphorylation of AKT at both S473 and T308, as well as AS160 at T642. Neither AICAR nor 991 had any effect on AKT phosphorylation under basal or insulin-stimulated conditions (Figure 4a–c). Similarly, 991 had no effect on basal or insulin-stimulated AS160 T642 phosphorylation, although the significant stimulatory effect of insulin was lost upon preincubation with AICAR for 24 h (Figure 4a,d).

### 3.4. AICAR but Not 991 Reduces Insulin-Stimulated MEK1/2-ERK1/2 Signalling in 3T3-L1 Adipocytes

**Figure 5:**
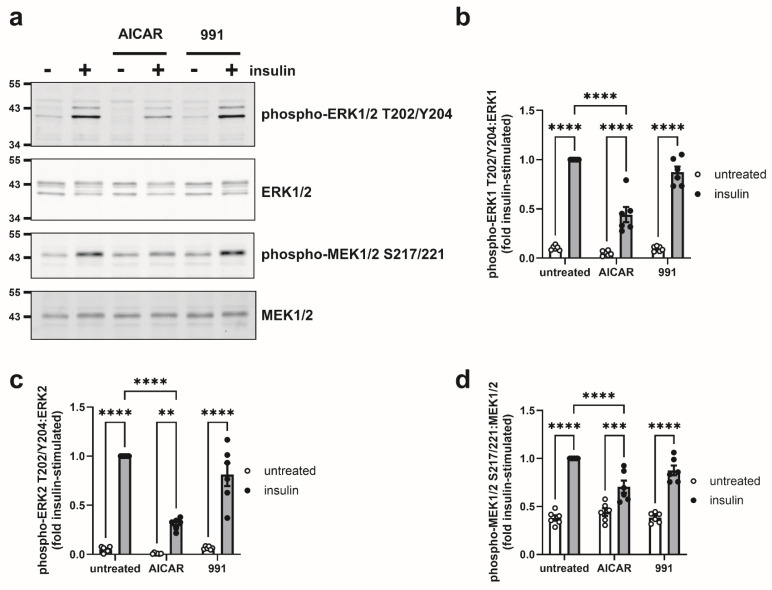
Effect of AICAR and 991 on MEK1/2 and ERK1/2 phosphorylation in 3T3-L1 adipocytes. 3T3-L1 adipocytes were stimulated in the presence or absence of 1 mM AICAR or 5 µM 991 for 24 h prior to the presence or absence of insulin (100 nM, 15 min). Lysates were prepared and immunoblotted with the antibodies indicated. (**a**) Representative immunoblots are shown with the indicated molecular masses in kDa. (**b**–**d**) Quantification of (**b**) ERK1 T202/Y204 phosphorylation normalised to ERK1, (**b**) ERK2 T202/Y204 phosphorylation normalised to ERK2, (**d**) MEK1/2 S217/221 phosphorylation normalised to MEK1/2 from six independent experiments (** *p* < 0.01, *** *p* < 0.001, **** *p* < 0.0001).

Insulin increases Ras-GTP levels, stimulating Raf to phosphorylate and activate MEK1/2, which in turn phosphorylates and activates ERK1/2 [1]. Insulin-stimulated ERK1/2 activation has been hypothesised to be important during adipogenesis [34], and ERK1/2 inhibition was reported to inhibit insulin-stimulated glucose uptake and improve insulin sensitivity in 3T3-L1 adipocytes [35,36]. Furthermore, AICAR has been reported to inhibit cytokine-stimulated ERK1/2 phosphorylation in 3T3-L1 adipocytes [37]. Therefore, the effect of AICAR and 991 on insulin-stimulated MEK1/2-ERK1/2 phosphorylation was assessed. Insulin significantly increased MEK1/2 and ERK1/2 phosphorylation in 3T3-L1 adipocytes. Preincubation with AICAR (1 mM) for 24 h markedly inhibited activating phosphorylation of both MEK1/2 and ERK1/2, whereas 991 (5 µM) had no effect (Figure 5). Neither AICAR nor 991 had any significant effect on basal MEK1/2 or ERK1/2 phosphorylation.

### 3.5. Adenosine Kinase Inhibitors Inhibit the Effect of AICAR on Insulin-Stimulated Glucose Uptake

**Figure 6:**
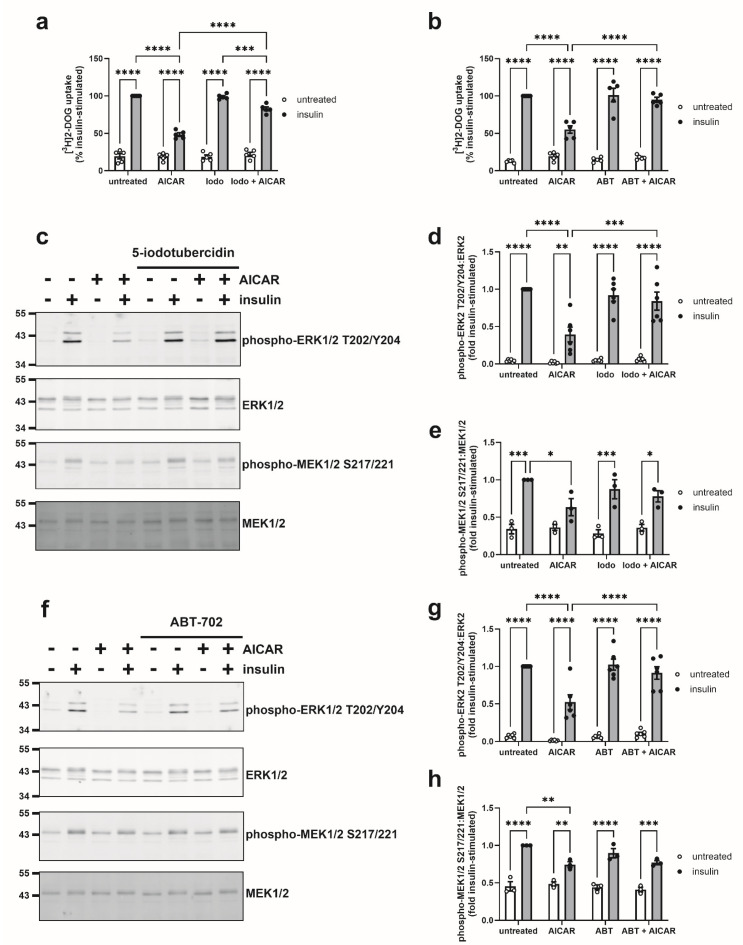
Inhibition of adenosine kinase reverses the inhibitory effect of AICAR on glucose uptake and ERK1/2 phosphorylation in 3T3-L1 adipocytes. 3T3-L1 adipocytes were stimulated in the presence or absence of 1 mM AICAR and/or (**a**,**c**–**e**) 0.1 µM 5-iodotubercidin (Iodo) or (**b**,**f**–**h**) 0.5 μM ABT-702 (ABT) for 24 h prior to the presence or absence of insulin (100 nM, 15 min). (**a**,**b**) [^3^H]2-deoxyglucose (DOG) uptake was assessed, and data presented % insulin-stimulated uptake from five independent experiments. (**c**–**h**) Lysates were prepared and immunoblotted with the antibodies indicated. (**c**,**f**) Representative immunoblots are shown with the indicated molecular masses in kDa. (**d**,**e**,**g**,**h**) Quantification of (**d**,**g**) ERK2 T202/Y204 phosphorylation normalised to ERK2, (**e**,**h**) MEK1/2 S217/221 phosphorylation normalised to MEK1/2 from six or three independent experiments, respectively (* *p* < 0.05, ** *p* < 0.01, *** *p* < 0.001, **** *p* < 0.0001).

AICAR phosphorylation to ZMP is catalysed by adenosine kinase. To examine whether the inhibitory effects of AICAR required its intracellular conversion to ZMP, cells were preincubated with two potent adenosine kinase inhibitors, 5-iodotubercidin and ABT-702. Neither 5-iodotubercidin (0.1 μM) nor ABT-702 (5 µM) had any effect on basal or insulin-stimulated glucose uptake, yet both significantly attenuated the inhibition of insulin-stimulated glucose uptake by AICAR (Figure 6a,b). Furthermore, both inhibitors had no effect on basal or insulin-stimulated ERK2 or MEK1/2 phosphorylation but significantly attenuated the inhibition of insulin-stimulated ERK2 phosphorylation by AICAR (Figure 6d,g). The effects of both inhibitors on ERK2 phosphorylation were associated with a loss of the significant effect of AICAR on insulin-stimulated MEK1/2 phosphorylation (Figure 6e,h).

### 3.6. Inhibition of MEK1/2 Does Not Phenocopy the Effect of AICAR on Insulin-Stimulated Glucose Uptake

**Figure 7:**
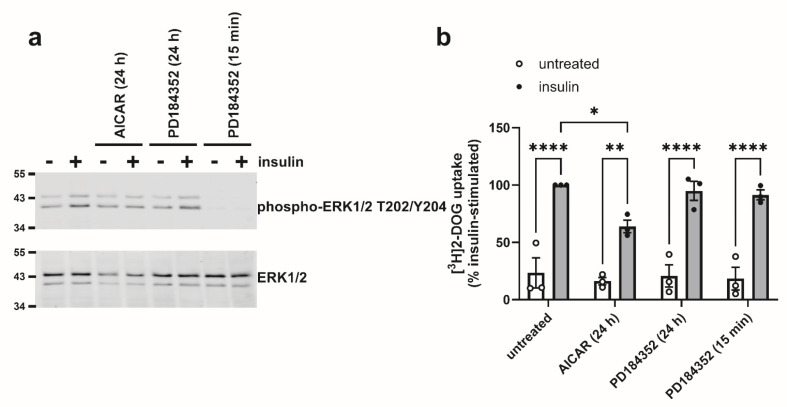
Effect of MEK inhibition on [^3^H]2-deoxyglucose uptake in 3T3-L1 adipocytes. 3T3-L1 adipocytes were stimulated in the presence or absence of 1 mM AICAR or 3 µM PD184352 for the durations indicated prior to the presence or absence of insulin (100 nM, 15 min). (**a**) Lysates were prepared and immunoblotted with the antibodies indicated. Representative immunoblots are shown with the indicated molecular masses in kDa from three independent experiments. (**b**) [^3^H]2-deoxyglucose (2-DOG) uptake was assessed. Data shown represent % insulin-stimulated [^3^H]2-DOG uptake from three independent experiments (* *p* < 0.05, ** *p* < 0.01, **** *p* < 0.0001).

Given the association between AICAR-mediated inhibition of insulin-stimulated glucose uptake and ERK1/2 phosphorylation, the effect of the MEK1/2 inhibitor PD184532 was assessed on insulin-stimulated glucose uptake, to determine whether this phenocopied AICAR. Surprisingly, incubation of 3T3-L1 adipocytes with PD184352 (3 µM) for 24 h had no effect on basal and insulin-stimulated ERK1/2 phosphorylation, yet preincubation for 15 min suppressed basal and insulin-stimulated ERK1/2 phosphorylation (Figure 7a). Preincubation with PD184352 for either time had no effect on basal or insulin-stimulated glucose uptake (Figure 7b).

### 3.7. Insulin-Stimulated Glucose Uptake and GLUT4 Levels Are Unaltered in Adipocytes of AMPKα1 KO Mice

**Figure 8:**
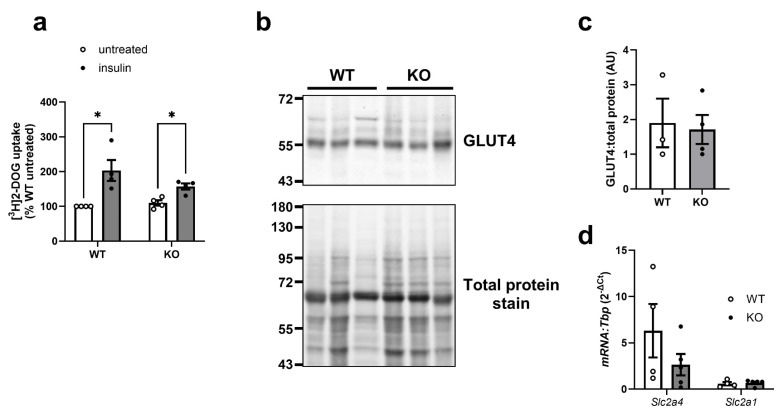
Insulin-stimulated glucose uptake and GLUT4 levels are unaltered in gonadal adipose tissue of AMPKα1 KO mice. (**a**) Adipocytes were prepared from female gonadal adipose tissue of WT or AMPKα1 KO mice and stimulated with 10 nM insulin for 30 min prior to assessment of [^3^H]2-DOG uptake. Data shown represent % WT untreated adipocyte 2-DOG uptake from four independent experiments, * *p* < 0.05 (2-way ANOVA with Fisher’s LSD test) (**b**–**d**) Epididymal adipose tissue was isolated from 20 week old WT or AMPKα1 KO mice and (**b**,**c**) lysates prepared and immunoblotted with anti-GLUT4 antibodies or (**d**) levels of *Slc2a4* and *Slc2a1* mRNA expression analysed by qPCR. (**b**) A representative immunoblot and total protein stain are shown with the indicated molecular masses in kDa. (**c**) Quantification of GLUT4 level normalised to REVERT total protein stain (arbitrary units) from three (WT) or four (AMPKα1 KO) mice. (**d**) Data shown represent mRNA expression in four (WT) or five (AMPKα1 KO) mice, normalised to *Tbp* (2^−ΔCt^).

We have previously demonstrated that perivascular adipose tissue function is impaired in AMPKα1 KO mice [27,38,39]. Furthermore, adipocytes have been reported to be smaller in epididymal and inguinal adipose tissue of AMPKα1 KO mice, associated with increased lipolysis [28]. As AMPKα1 is the catalytic isoform responsible for most total cellular AMPK activity in adipocytes [18,28,29], we examined whether sustained suppression of AMPK influenced adipocyte glucose uptake in gonadal adipocytes from female mice. Insulin stimulated 2-deoxyglucose uptake in adipocytes from both WT and AMPKα1 KO mice, yet neither basal nor insulin-stimulated glucose uptake was affected by genotype (Figure 8a). Furthermore, levels of GLUT4 mRNA or protein in epididymal adipose tissue were unaffected by genotype (Figure 8b–d). As AICAR increased *Slc2a1* mRNA levels in 3T3-L1 adipocytes (Figure S1b), the effect of AMPKα1 KO on *Slc2a1* mRNA levels was assessed. Genotype had no effect on epididymal adipose tissue *Slc2a1* levels (Figure 8d).

## 4. Discussion

We and others have previously reported that AICAR rapidly reduced insulin-stimulated glucose uptake in both 3T3-L1 adipocytes and isolated rodent adipocytes [11,18,19]. The current study extends these observations, demonstrating that prolonged stimulation with AICAR markedly inhibits insulin-stimulated glucose uptake without affecting basal glucose uptake in 3T3-L1 adipocytes. The mechanism by which prolonged AICAR suppresses insulin-stimulated glucose uptake is (1) likely to be AMPK-independent; (2) ZMP-dependent; (3) not associated with altered GLUT4 levels, and (4) associated with impaired insulin-stimulated GLUT4 translocation and MEK1/2-ERK1/2 phosphorylation but not AKT or AS160 T642 phosphorylation.

The AMPK-independence of the inhibition of insulin-stimulated glucose uptake by AICAR is supported by the direct AMPK activator 991 having no effect on glucose uptake or GLUT4 translocation in 3T3-L1 adipocytes despite stimulating AMPK to a similar extent. These data agree with previous work in which 991 had no rapid effect on glucose uptake in 3T3-L1 adipocytes or rodent adipocytes [11]. In another study, insulin-stimulated glucose uptake in mouse epididymal fat pads was reduced by coincubation with AICAR and the direct activator SC4. This effect was likely AMPKα1-independent, as the inhibitory effect was still observed in fat pads from AMPKα1 KO mice [25]. The 991 shows some selectivity for AMPK complexes containing the β1 subunit isoform over those containing the β2 isoform [40,41]. We have previously reported that complexes containing AMPKβ2 account for most total cellular activity in 3T3-L1 and rodent adipocytes [29], although this has been disputed [42]. Regardless of which AMPKβ isoforms predominate in adipocytes, it is unlikely that the lack of effect of 991 is due to isoform selectivity, as the concentration used was well above the in vitro half maximal concentration (~1 µM) for complexes containing AMPKβ2 [40], and a similar degree of ACC phosphorylation was observed with AICAR and 991. Taken together, these data indicate that the effect of AICAR is AMPK-independent, although AICAR-mediated activation of specific pools of AMPK that are not activated by 991 cannot be completely excluded. Previous studies have reported that incubation with AICAR for shorter times (up to 2 h) stimulated basal glucose uptake in 3T3-L1 adipocytes [18,43,44,45,46], yet this is not observed in isolated rodent and human adipocytes [11,19] and was reported to be AMPK-independent in 3T3-L1 adipocytes [43]. As ZMP may mimic AMP, this raises the question as to whether metabolic stress and the subsequent increase in AMP may increase basal glucose uptake to meet the energetic demands of adipocytes, whilst reducing insulin-stimulated glucose uptake for lipogenesis that would consume ATP [11]. Others have reported that the mitochondrial uncoupler dinitrophenol inhibits ATP production and increases basal glucose uptake in both 3T3-L1 adipocytes and isolated adipocytes [44,46,47]. Stimulation with AICAR for up to 1 h activates AMPK without altering adenine nucleotide ratios in hepatocytes [6] or 3T3-L1 adipocytes [48], yet whether prolonged AICAR stimulation altered adenine nucleotide ratios in the current study was not examined. Prolonged stimulation with AICAR had no effect on basal glucose uptake, in agreement with a previous study [49]. Although it remains possible that agents that alter adenine nucleotides increase basal uptake acutely, these data demonstrate that prolonged stimulation with AICAR has no effect on basal glucose uptake. This is reinforced by glucose uptake measurements in adipocytes from AMPKα1, where basal and insulin-stimulated glucose uptake was unaltered, suggesting that sustained suppression of AMPK also has no effect on glucose uptake in adipocytes. It should be noted that this study only examined female gonadal adipose tissue, yet others have reported no change in glucose uptake in isolated gonadal adipose or in vivo in male mice lacking AMPK [24,25].

Prolonged stimulation with AICAR or 991 also had no effect on insulin signalling via AKT-AS160. This agrees with previous work with shorter AICAR incubation [11,18] yet contrasts with one report where insulin-stimulated AS160 T642 phosphorylation was reduced by AICAR (2 mM for 1 h) in 3T3-L1 adipocytes, without any effect on AKT [20]. The inhibition of insulin-stimulated glucose uptake by AICAR was, however, associated with markedly reduced GLUT4 translocation to the plasma membrane as assessed with tagged GLUT4, similar to previous data using a plasma membrane lawn technique or subcellular fractionation after AICAR incubation for a short period [18,20]. Intriguingly, AICAR suppressed insulin-stimulated phosphorylation of MEK1/2 and its substrate ERK1/2 in agreement with previous studies in human endothelial cells [50] and insulin-like growth factor-1-stimulated 3T3-L1 preadipocytes [51]. In contrast, 991 had no effect, indicating another AMPK-independent action of AICAR. A previous study has reported that dinitrophenol and azide, which would increase the AMP:ATP ratio, impaired insulin-stimulated AKT and ERK1/2 phosphorylation in 3T3-L1 adipocytes [47]. The latter is consistent with the effect of ZMP in the current study, whereas the effect on AKT was not observed and could reflect the greater metabolic stress in response to dinitrophenol or azide. Furthermore, ERK1/2 inhibition has been reported to improve insulin sensitivity and inhibit insulin-stimulated glucose uptake in 3T3-L1 adipocytes [35,36]. In contrast, inhibition of MEK1/2 with PD184532 in the current study had no rapid effect on insulin-stimulated glucose uptake. The reason for the discrepancy between these data remains uncertain, yet the lack of effect of PD184532 indicates that suppression of insulin-stimulated MEK1/2-ERK1/2 activation does not underlie the inhibitory action of AICAR on glucose uptake.

Prolonged stimulation with AICAR or 991 had no effect on GLUT4 levels in 3T3-L1 adipocytes, in agreement with a previous study using 2 mM AICAR for 24 h [49]. GLUT4 levels were also unaffected in adipose tissue of mice lacking AMPKα1, indicating AMPK does not regulate GLUT4 levels, despite previous studies indicating AICAR increases muscle GLUT4 expression via AMPK-mediated phosphorylation of GEF and/or HDAC5 [22,23]. Both GEF and HDAC5 are expressed in mouse adipose, and HDAC5 is inversely associated with GLUT4 levels during adipocyte differentiation [52], yet the current study indicates that any effect of AICAR/AMPK on GLUT4 levels is limited to muscle. AICAR, but not 991, did increase levels of mRNA encoding GLUT1 in 3T3-L1 adipocytes. AMPK activation has been reported to increase GLUT1-mediated glucose uptake by a mechanism involving TXNIP regulating GLUT1 gene expression, stability and localisation [53]. The effect of AICAR in the current study is consistent with this, but the lack of effect of 991 again suggests an AMPK-independent action of AICAR on GLUT1 gene transcription. Given the lack of effect of AICAR on basal glucose uptake and suppression of insulin-stimulated glucose uptake, it is unlikely that this is mediated by increased GLUT1, which would be predicted to improve, not suppress glucose uptake.

Two different inhibitors of adenosine kinase ablated the effect of AICAR on insulin-stimulated glucose uptake and ERK1/2 phosphorylation, demonstrating that the effect of AICAR is ZMP-dependent. ZMP has been previously demonstrated to mimic the effects of AMP on enzymes such as GP and FBP1 that are allosterically regulated by AMP [8,9]. AICAR would therefore be expected to stimulate glycolysis due to increased phosphofructokinase activity and reduced FBP1 activity via the allosteric actions of ZMP, so this cannot underlie the observed effects of AICAR on 2-deoxyglucose uptake. Mitochondrial glycerophosphate dehydrogenase has also been demonstrated to be inhibited by AMP [54]. AICAR might therefore be expected to reduce glycerol production, but this would not likely be linked to GLUT4 translocation. Insulin-stimulated GLUT4 translocation and MEK1/2-ERK1/2 phosphorylation do require activation of small G-proteins (the Rab family and Ras, respectively), so future studies should examine whether these are sensitive to ZMP/AMP, and this underlies the effect of AICAR. Indeed, AICAR has been reported to suppress IGF-1-stimulated GTP-Ras levels in NIH-3T3 cells [51] and prolonged AICAR-depleted GTP levels in CHO-K1 cells [55]. Inhibition of AICAR transformylase, which increases ZMP, also increased GMP levels in mouse tumours [56]. These studies suggest ZMP may increase the GMP:GTP ratio, which could suppress small G-protein activation. Alternatively, ZMP/AMP could affect other components of the GLUT4 trafficking pathway downstream of AS160 or parallel pathways that have been demonstrated to be required for insulin-stimulated GLUT4 translocation, such as the small G-protein TC10 [57].

Therefore, AICAR inhibits insulin-stimulated glucose uptake via an as yet uncharacterised mechanism that requires conversion to ZMP. This suggests that adenine nucleotide levels and, therefore, energy charge can regulate adipocyte glucose uptake independently of AMPK. This may be of importance in regulating adipocyte glucose uptake when cellular energy reserves are low, suppressing anabolic triglyceride production and sparing ATP for other purposes. At the same time, reduced energy charge would increase AMPK activity, which would suppress lipogenesis and thereby conserve ATP further [4].

## Data Availability

The raw data supporting the conclusions of this article will be made available by the authors on request.

## Supplementary Materials

The following supporting information can be downloaded at: https://www.mdpi.com/article/10.3390/cells14221811/s1. Supplementary Figure S1: Effect of 2mM AICAR on [^3^H]2-deoxyglucose uptake and GLUT gene expression in 3T3-L1 adipocytes.

## Abbreviations

The following abbreviations are used in this manuscript:

2-DOG	2-deoxyglucose
3T3-HA-GLUT4-GFP	preadipocytes stably expressing HA-GLUT4-GFP
ACC	acetyl-CoA carboxylase
AICAR	5-Aminoimidazole-4-carboxamide ribonucleoside
AKT	protein kinase B
AMPK	AMP-activated protein kinase
AS160	AKT substrate of 160 kDa/TBC1D4
ERK	extracellular signal-regulated kinase
FBP1	fructose 1,6-bisphosphatase
GEF	GLUT4 enhancer factor
GLUT	glucose transporter
GP	glycogen phosphorylase
HDAC	histone deacetylase
HK	hexokinase
IBMX	3-Isobutyl-1-methylxanthine
KO	knockout
MEF	myocyte enhancer factor
MEK	mitogen-activated protein kinase kinase
*Slc2a1*	gene encoding GLUT1
*Slc2a4*	gene encoding GLUT4
*Tbp*	gene encoding TATA binding protein
WT	wild type
ZMP	5-aminoimidazole-4-carboxamide-ribofuranosyl 5′-phosphate
