# Supplementary material for: AICAR Inhibits Insulin-Stimulated Glucose Uptake in 3T3-L1 Adipocytes via an AMPK-Independent, ZMP-Dependent Mechanism
Source: Cells. 2025 Nov 18;14(22):1811. doi: 10.3390/cells14221811 (PMC12651217; doi:10.3390/cells14221811)
Supplement: Supplementary file 1 [file cells-14-01811-s001.zip › cells-3936180-supplementary.pdf]

cells

MDPI

Article

AICAR Inhibits Insulin-Stimulated Glucose Uptake in 3T3-L1 Adipocytes via an AMPK-Independent, ZMP-Dependent Mechanism

Yazeed Alshuweishi [1,2], Fatmah Binzomah Alghamdi [1,3], Kieran Patrick [1] and Ian P. Salt [1,*]

1 School of Molecular Biosciences, College of Veterinary, Medical and Life Sciences, University of Glasgow, Glasgow G12 8QQ, UK; yalshuweishi@ksu.edu.sa (Y.A.); fsaalghamdi7@kau.edu.sa (F.B.A.); kieranpatrick47@gmail.com (K.P.
2 Department of Clinical Laboratory Sciences, King Saud University, Riyadh 11433, Saudi Arabia
3 Department of Clinical Pharmacology, Faculty of Medicine, King Abdulaziz University, Jeddah 21589, Saudi Arabia
* Correspondence: ian.salt@glasgow.ac.uk

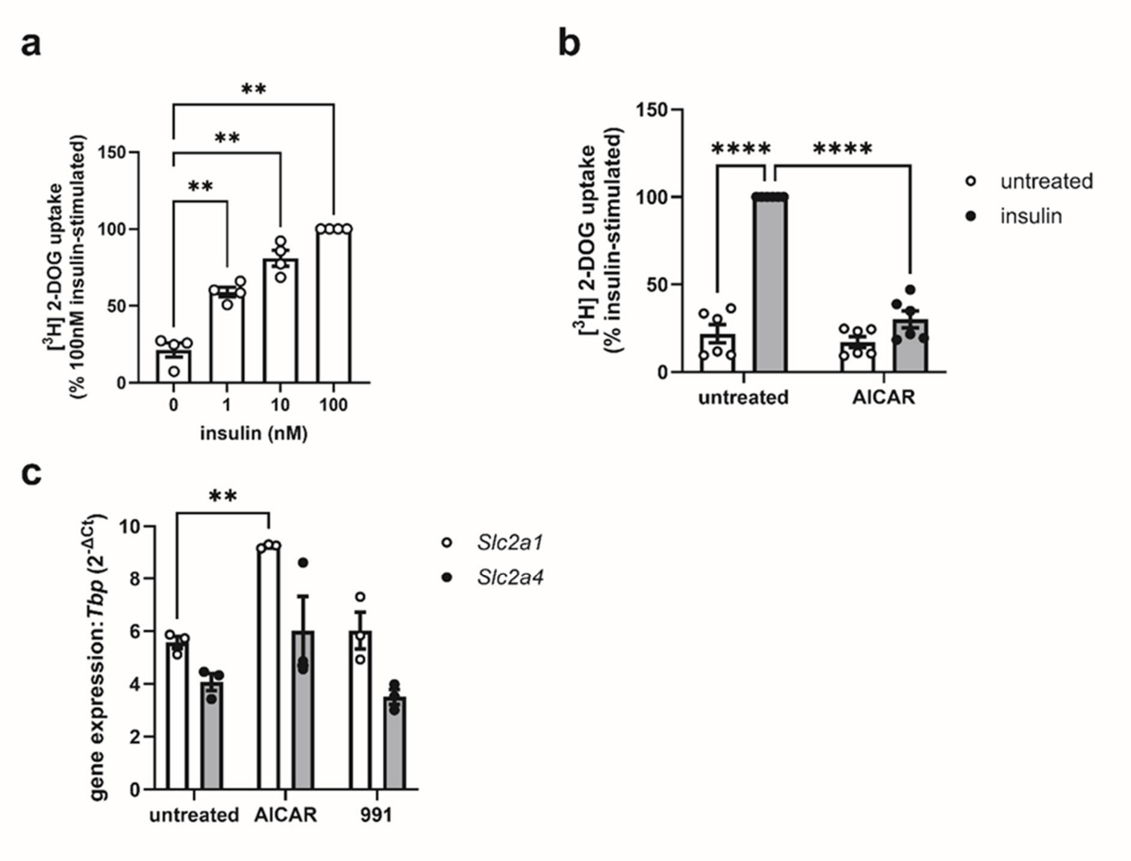

Supplemental Figure 1. Effect of 2 mM AICAR on [^{3}H]2-deoxyglucose uptake and GLUT gene expression in 3T3-L1 adipocytes. 3T3-L1 adipocytes were (**a**) stimulated in the indicated concentrations of insulin for 15 min or (**b**) incubated in the presence or absence of 2 mM AICAR or 5 µM 991 for 48 h prior to incubation in the presence or absence of insulin (100 nM, 15 min) and [^{3}H]-2-deoxyglucose ([^{3}H]2-DOG) assessed. The mean ± SEM % insulin-stimulated [^{3}H]2-DOG uptake from (**a**) four or (**b**) six independent experiments is shown. (**c**) 3T3-L1 adipocytes were stimulated in presence

 https://doi.org/10.3390/cells14221811

or absence of 2 mM AICAR or 5 μM 991 for 48 h, RNA isolated and levels of *Slc2a1* and *Slc2a4* mRNA expression analysed by qPCR. Data shown represents mRNA expression normalised to *Tbp* from three independent experiments ($2^{-\Delta Ct}$). **p<0.01, ****p<0.0001 (**a**) 1-way ANOVA with Holm-Šídák's multiple comparisons test or (**b,c**) 2-way ANOVA with Šídák's multiple comparisons test.
